# The Effects of Resistance Exercise on Muscle Damage, Position Sense, and Blood Redox Status in Young and Elderly Individuals

**DOI:** 10.3390/geriatrics2030020

**Published:** 2017-06-23

**Authors:** Michalis G. Nikolaidis

**Affiliations:** Department of Physical Education and Sports Sciences at Serres, Aristotle University of Thessaloniki, Agios Ioannis, 62110 Serres, Greece; nikolaidis@auth.gr; Tel.: +30-231-099-1086; Fax: +30-232-106-4806

**Keywords:** delayed onset muscle soreness, glutathione, isokinetic dynamometry, protein carbonyls, range of motion

## Abstract

*Background:* The purpose of the present investigation was to study the possible differences between young and elderly individuals regarding muscle damage, position sense, and oxidative stress biomarkers in response to resistance eccentric-biased exercise. *Methods:* Ten young and 10 elderly individuals performed a bout of resistance exercise (i.e., squat exercise). Muscle damage indices (i.e., isometric peak torque, range of movement, delayed onset muscle soreness, and creatine kinase), position sense, and oxidative stress biomarkers (i.e., protein carbonyls and reduced glutathione) were assessed before and 48 h post exercise. *Results:* The main effect of time was significant for all measured parameters, indicating that resistance exercise that includes a large eccentric component causes muscle damage, disturbs position sense, and induces oxidative stress. However, no significant main effect of group or time × group interaction was found for all measured parameters (except isometric peak torque), indicating similar responses to resistance exercise for both young and the elderly individuals. *Conclusion:* There are no differences between young and elderly individuals regarding muscle damage, position sense, and oxidative stress after resistance exercise, while elderly individuals have lower muscle strength and seem to have a tendency for greater baseline oxidative stress compared to young individuals.

## 1. Introduction

It is known that human skeletal muscle mass starts to decrease by 1–2% per year by the age of 30 years [1], a condition that renders older individuals vulnerable to develop sarcopenia (i.e., muscle mass decrease). Sarcopenia is a key factor contributing to frailty, loss of functional mobility, and loss of independence [2], as well as mortality in the elderly [3]. On the other hand, it has been suggested that strength training in senescence can improve the function and morphology of human skeletal muscle [4], and in sequence, delay muscle loss.

Typically, individuals over the age of 55 years rarely participate in moderate and vigorous physical activities, while it is generally established that physical inactivity is strongly associated with ageing [5,6]. Taking into account these considerations, in a previous study, we used an isokinetic dynamometer to perform resistance exercise, and significant improvements in health risk factors were found (i.e., improvements in resting energy expenditure and fat oxidation, improvements in lipid profile and insulin sensitivity) [7]. However, the use of isokinetic dynamometer for resistance exercise employs a rather non-physiological muscle movement (i.e., pure eccentric actions of knee extensors) while at the same time the exercise protocol is conducted with maximal effort that is difficult to be followed by the elderly. Considering these limitations, in order to investigate the effects of demanding daily activities on muscle damage, position sense, and redox status, the squat movement was employed. The squat movement provides extensive loads of eccentric (during downward movement) and concentric (during upward movement) muscle actions, while it also mimics a number of daily human movements (i.e., standing up from a chair or sitting on a chair).

An eccentric muscle action is used when the muscle lengthens to lower a load (e.g., sitting on a chair) and, generally, an eccentric action occurs when the muscle unsuccessfully resists elongation, acting as a brake. However, lengthening the muscle during eccentric actions may lead to muscle damage [8], and is characterized among others by sustained loss of muscle strength, range of motion (ROM), development of delayed onset muscle soreness (DOMS), and increases in the concentration of creatine kinase (CK) in the blood [7,9,10,11]. These changes typically begin approximately 6 h after unaccustomed exercise and peak at one to three days [12,13,14]. Moreover, in previous studies the effect of pure eccentric exercise on the position sense of the legs was examined [13,15,16]. It is a common experience that people face difficulty in performing daily movements after activities that require lowering the body by bending the knee (i.e., squat movement). This situation may relate to the diminished position sense of the lower limbs and may increase the risk for injuries. Indeed, in a previous investigation of our group, it was found that muscle-damaging eccentric exercise led subjects to adopt a more extended position of their limbs [13]. A possible mechanism could be the disrupted sarcomeres that led to an increase of the muscle’s series compliance, causing the limbs to adopt a shorter muscle length (i.e., a more extended position of the limb) [17]. However, there is a lack of literature regarding the effects of squat exercises on position sense in elderly individuals.

Resistance exercise, which includes a large eccentric component, also induces alterations in redox status that are characterized by increases in oxidant biomarkers (lasting for up to four days after exercise and even up to 40% compared to rest) [18,19,20,21,22]. This characteristic of resistance exercise permits the investigators to monitor the long and large alterations of redox biomarkers in responses to resistance exercise. Given the fact that redox biology processes can augment muscle damage by oxidative modifications to the contractile machinery proteins, then, in turn, they also influence position sense. On this basis, it is interesting to investigate whether redox biomarkers respond similarly with muscle damage indices and position sense after squat movement in young and elderly individuals. Based on the fact that the elderly age group is a much less frequently investigated population on the subject of unaccustomed exercise, compared to young individuals [23], the aim of the present investigation was to compare muscle damage, position sense, and redox biomarkers of young and elderly individuals in response to resistance exercise.

## 2. Results

**Figure 1:**
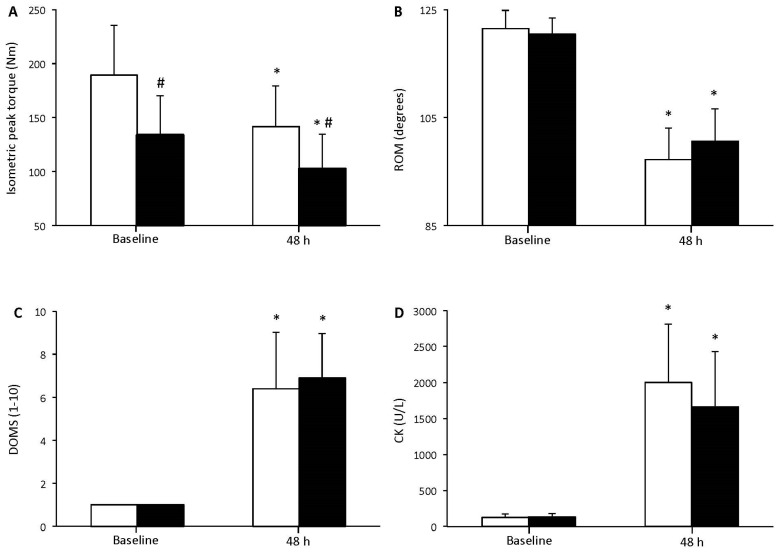
Isometric peak torque at 90° knee flexion (**A**); free-pain range of movement (**B**); delayed onset muscle soreness (**C**); and creatine kinase CK (**D**) before and 48 h after resistance exercise for young (white bars) and elderly (black bars) individuals (mean ± SD). * indicates a significant difference compared to baseline in the same group. # indicates a significant difference between the two groups at the same time point.

**Figure 2:**
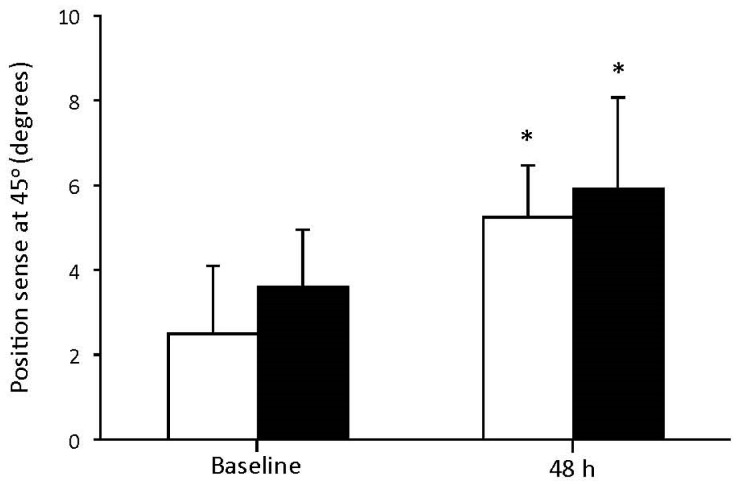
Position sense at 45° before and 48 h after resistance exercise for young (white bars) and elderly (black bars) individuals (mean ± SD). * indicates a significant difference compared to baseline in the same group.

There were significant differences in physical characteristics between young and elderly individuals. More specifically, young participants were lighter, and exhibited lower body fat and higher fat free mass (*p* < 0.05) compared to the elderly individuals. In isometric peak torque, ROM, DOMS, and CK, the main effect of time was found to be significant (*p* < 0.001; Figure 1A–D), while significant alterations of isometric peak torque, ROM, DOMS, and CK were found at 48 h post resistance exercise. Regarding isometric peak torque, there was a significant main effect of group indicating lower muscle strength in elderly compare to young individuals at baseline and 48 h post resistance exercise. Regarding ROM, DOMS, and CK, there was no significant main effect of group or time × group interaction. In position sense at 45°, the main effect of time was found to be significant (*p* < 0.001; Figure 2), while position sense disturbances were observed at 48 h post resistance exercise in both groups. No significant main effect of group or time × group interaction was found in position sense.

**Figure 3:**
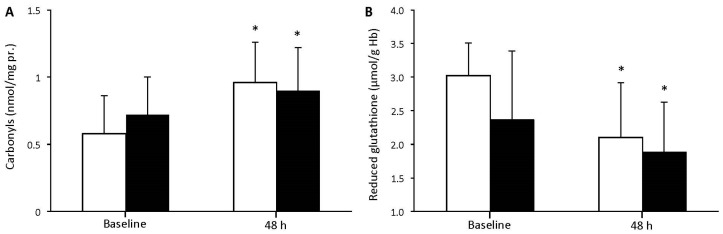
Protein carbonyls (**A**) and reduced glutathione (**B**) before and 48 h after resistance exercise for young (white bars) and elderly (black bars) individuals (mean ± SD). * indicates a significant difference compared to baseline in the same group.

There was no significant main effect of group or time × group interaction concerning protein carbonyls (Figure 3A) and reduced glutathione (GSH) (Figure 3B). However, there was a significant main effect of time (*p* < 0.001) for both redox status indices, showing for each one of the young and elderly groups significant alterations 48 h after resistance exercise compared to baseline values.

## 3. Discussion

The purpose of the present investigation was to study the possible differences between young and elderly individuals regarding muscle damage, position sense, and oxidative stress biomarkers in response to a commonly used resistance exercise (namely squat movement). Resistance exercise increased muscle damage and disturbed position sense to a similar degree between young and elderly individuals. As was expected, the muscle strength of the elderly was significantly lower compared to young individuals at baseline. Regarding oxidative stress, the older individuals showed a tendency for greater baseline oxidative stress compared to the young individuals (i.e., increased protein carbonyls and decreased GSH). However, in both groups, oxidative stress biomarkers changed similarly after resistance exercise.

It was clear in the present investigation that there was no effect of age at baseline values regarding range of motion and muscle soreness. In contrast, there was significantly greater isometric peak torque in the young compared to older participants at baseline and 48 h post resistance exercise. After the acute bout of eccentric exercise, comparable alterations appeared in muscle damage markers in both age groups, suggestive of comparable muscle damage in young and elderly individuals. The present results are in line with a part of the available literature that supports similar increases in muscle damage between young and elderly individuals that performed eccentric exercise using resistance-training equipment [23,24]. However, the present data disagree with another part of the literature supporting the idea that older adults have higher levels of muscle damage after a single bout of unaccustomed exercise than young adults [25,26,27]. In previous investigations, the use of isokinetic contractions during eccentric exercise [25] and the use of female participants [26,27] could partially explain the observed discrepancies between the present findings with the previous ones.

It was also found that resistance exercise disturbed position sense, since individuals of both groups after resistance exercise placed their limb in a more extended position relative to the reference angle (i.e., 45°). The most relevant studies have measured position sense after eccentric exercise of the legs using isokinetic dynamometer and have also reported disturbances to position sense at the knee joint [13,15,16]. The present data could be explained by the fact that signals from muscle spindles contribute to the sense of position and movement of the limbs [28], and it has been proposed that the rise in passive tension after resistance exercise that involves eccentric muscle actions can mechanically unload muscle spindles [29]. The unloading of muscle spindles can lower their passive discharge rates, leading subjects to adopt a position of their knee joint different from the target angle.

Intense resistance exercise was previously used as an experimental model to disturb redox homeostasis in a physiological manner [30,31,32]. However, in a number of investigations an acute bout of exercise failed to induce oxidative stress [33,34]. This is the reason why, in the present study, a resistance exercise that includes a large eccentric component (i.e., squat movement) was selected, which causes long (lasting up to four days after exercise) and large (even up to 40% compared to rest) increases in oxidant biomarkers [18,19,20,21,22]. A non-statistically significant trend for lower GSH and higher protein carbonyls values was found in the elderly compared to the young group. The lower levels of GSH could be partly explained by the scavenging of the aging-induced free radical production and its consumption to regenerate ascorbic acid [35]. In response to the acute eccentric exercise, a higher protein carbonyl concentration in plasma (a biomarker of generic protein oxidation [36]) was observed in young and elderly groups, indicating the occurrence of oxidative stress. As expected, the levels of GSH were lower after exercise in both groups, since GSH represents the most important low-molecular weight non-enzymatic antioxidant of erythrocytes. More specifically, it serves both as a free radical scavenger and as a reductive substrate for other enzymatic (e.g., glutathione peroxidase; GPx) and non-enzymatic (e.g., vitamin C) antioxidants [37].

It is known that aging causes reductions in muscle mass and strength [38], which is also supported by the findings of the present investigation, while sarcopenia is a major factor contributing to decreased functional independence and mobility [2]. Moreover, impaired muscle strength in the elderly is associated with the increased risk of functional impairments [39], leading to decreased independence and mobility [2]. It is clear that in elderly individuals a moderate level of muscle strength is required in order to maintain autonomy in daily living [40]. It is concluded that after resistance exercise, the elderly individuals experience similar muscle damage and position sense disturbances, while the alterations in redox homeostasis were comparable to their young counterparts. The slightly worse baseline oxidative stress observed in the group of elderly individuals did not affect their responses to resistance exercise.

## 4. Materials and Methods

### 4.1. Participants

Subjects were recruited after advertising the study in the local media. Ten young males (age 22.1 ± 3.9 years; weight 72.4 ± 7.5 kg; height 175 ± 6 cm; body mass index (BMI) 23.6 ± 2.4; body fat 13.5 ± 3.8%; free fat mass 62.5 ± 5.4 kg) and 10 elderly males (age 66.9 ± 5.4 years; weight 75.2 ± 6.9 kg; height 173 ± 5 cm; BMI 25.2 ± 2.2; body fat 28.6 ± 7.5%; free fat mass 53.6 ± 6.0 kg) participated in the study. Subjects were untrained and were excluded from the study if they had participated in scheduled resistance exercise or other activities with a large eccentric component (i.e., downhill walking or running and intense plyometric exercises) for at least six months before the study. Participants of both groups could be considered of the same physical activity level since they participated in low-intensity leisure activities (such as walking, swimming, and dancing) two to three times per week for less than 3 h·w^−1^. They were instructed to abstain from any exercise for three days before and during data collection, and were not taking anti-inflammatory drugs. Exclusion criteria included smoking. The study was carried out following the rules of the Declaration of Helsinki of 1975 as revised in 2008. Subjects read and signed an informed consent form after they were informed of all the risks, discomforts, and benefits involved in the study. Approval was received from the Research Ethics Committee of the European University Cyprus (016/12-05-2013).

### 4.2. Assessment of 1 Repetition Maximum (1RM)

Maximal squat strength was evaluated two to three days before the experimental exercise protocol using a plate-loaded Smith press (Technogy, Bracknell, UK). After a short warm-up on a cycle ergometer and stretching exercises of the major muscle groups, subjects performed incremental submaximal efforts until they were unable to lift a heavier load. Before the assessment, subjects performed an 8-min warm-up consisting of cycling on a Monark cycle ergometer (Monark, Vansbro, Sweden) at 70 rpm and 50 W, followed by 10 min of stretching exercises.

### 4.3. Research Design

All measurements were performed between 08:00 and 09:00 h. Body mass was measured to the nearest 0.5 kg (Beam Balance 710, Seca, Birmingham, UK) with subjects lightly dressed and barefoot. Standing height was measured to the nearest 1 cm (Stadiometer 208, Seca). Percentage body fat was calculated (the Siri skinfold equation was used) from seven skinfold measures (average of two measurements of the right site), using a Harpenden caliper (John Bull, West Sussex, UK). All participants undertook a resistance exercise session including the squat movement, and the intensity was set at 75% of one repetition maximum (1RM). Isometric peak torque, pain-free ROM, DOMS during squat movement, CK concentration in blood, position sense, protein carbonyls, and GSH were evaluated before and 48 h post exercise. The timepoint of 48 h post exercise was chosen for evaluation because it has been previously found that at this timepoint, eccentric exercise causes the greatest alterations in the studied parameters [7,15,20,21]. Each subject was familiarized at least two days before the evaluation of 1RM. This familiarization procedure involved 4-6 squat movements at a very low intensity (i.e., 20% of 1RM). Moreover, during the familiarization the exact position on the Smith press of the 90° knee angle for each participant was recorded for the follow up measurements.

### 4.4. Resistance Exercise Protocol

The resistance exercise was performed using the same plate-loaded Smith press. During the squat movement, subjects from the upright position with the loaded bar on their shoulders moved downwards so that knee angle reaches 90° at the lowest position and then moved upwards to the upright position in order to complete one repetition. Every downward or upward movement lasted 2 s (i.e., 4 s to come back at the upright position) and the duration was kept constant using a metronome. During resistance exercise, two people were standing at the edges of the barbell in order to hold the barbell in case of emergency. A block was set at 93° knee flexion so the barbell would stop in order to avoid injuries and prevent hyperflexion of the knee joint. The exercise session consisted of five sets of 15 squat movements, while a 2-min rest interval was incorporated between sets. Before the exercise session, subjects performed an 8-min warm-up consisting of cycling on a Monark cycle ergometer (Vansbro, Sweden) at 70 rpm and 50 W, followed by 10 min of stretching exercises.

### 4.5. Muscle Damage Indices

The assessment of isometric peak torque at 90° knee flexion and the pain-free ROM were performed on an isokinetic dynamometer (Cybex Norm, Ronkonkoma, NY, USA). During the isometric peak torque assessment at 90° knee flexion, the average of the three best maximal voluntary contractions with the dominant leg was recorded. To ensure that the subjects provided their maximal effort, the measurements were repeated if the difference between the lower and the higher torque values exceeded 10%. There was a 2-min rest between isometric efforts. During the ROM evaluation, the investigator moved the calf at a very low angular velocity from full extension (0°) of the knee to the position where the subject felt any discomfort. The angle was recorded to indicate the end of the pain-free ROM. All participants were pain-free at full extension. Volunteers subjectively assessed DOMS during a squat movement (90° knee flexion). Perceived soreness was rated on a scale ranging from 1 (normal) to 10 (very sore) as described previously [13]. The blood collection procedure for the measurement of CK and the assay protocol are described below in the relevant sub-sections.

### 4.6. Position Sense

The isokinetic dynamometer was used for the evaluation of position sense, which was calibrated weekly according to the manufacturer’s instructions. The procedures of the position sense evaluation have been described previously [15]. Briefly, subjects sat upright on the isokinetic dynamometer (with the trunk tilted back at a 120° hip angle). All assessments were performed on the dominant limb while the angles were automatically recorded by the dynamometer. During determination of the perception of knee joint angle, the limb was moved from full extension (0°) to 90° knee flexion in order to familiarize subjects with the range of motion. Then the investigator positioned the limb at the reference angle (45°), maintained it for 10 s, and returned the lower limb to the initial position (90°). Afterwards, subjects were asked to remember where the reference position was (45°) and reproduce it from memory. Subjects actively moved their limb to the target angle and, when they were satisfied with the angle they had selected, they would hold it for about 2 s. The degrees deviating from the reference angle were recorded. Two efforts were performed and the best one was recorded. The leg moved from 0° to 90° and then to the target position and back to 90° prior to each of the two efforts.

### 4.7. Blood Collection

Before and 48 h after the exercise protocol, blood samples were drawn from a forearm vein. The blood was collected into tubes containing ethylenediaminetetraacetic acid (EDTA). The blood was centrifuged immediately at 1370 g for 10 min at 4 °C and the plasma was collected. The packed erythrocytes were lysed with 1:1 (*v*/*v*) distilled water, inverted vigorously, and centrifuged at 4000× *g* for 15 min at 4 °C. The plasma or erythrocyte lysate was collected in multiple aliquots, stored at −80 °C and thawed only once before analysis.

### 4.8. Assays

Plasma protein carbonyls and erythrocyte GSH were determined spectrophotometrically [22]. Specifically, protein carbonyls were determined by adding 50 μL of 20% trichloroacetic acid (TCA) to 50 μL of plasma or erythrocyte lysate (diluted 1:10). This mixture was incubated in an ice bath for 15 min and centrifuged at 15,000× *g* for 5 min at 4 °C. The supernatant was discarded, and 500 μL of 10 mM 2,4-dinitrophenylhydrazine (in 2.5 N HCL) for the sample or 500 μL of 2.5 N HCL for the blank was added in the pellet. The samples were incubated in the dark at room temperature for 1 h, with intermittent vortexing every 15 min, and were centrifuged at 15,000× *g* for 5 min at 4 °C. The supernatant was discarded, and 1 mL of 10% TCA was added, vortexed, and centrifuged at 15,000× *g* for 5 min at 4 °C. The supernatant was discarded, and 1 mL of ethanol–ethyl acetate (1:1 *v*/*v*) was added, vortexed, and centrifuged at 15,000× *g* for 5 min at 4 °C. This washing step was repeated twice. The supernatant was discarded, and 1 mL of 5 M urea (pH 2.3) was added, vortexed, and incubated at 37 °C for 15 min. The samples were centrifuged at 15,000× *g* for 3 min at 4 °C, and the absorbance was read at 375 nm. Calculation of protein carbonyl concentration was based on the molar extinction coefficient of dinitrophenylhydrazine.

GSH was measured according to a modified protocol of Reference [41], as modified and presented in detail in Reference [42]. Specifically, 20 μL of erythrocyte lysate was treated with 5% TCA mixed with 660 μL of 67 mM sodium potassium phosphate (pH 8.0) and 330 μL of 1 mM 5,5’-dithiobis-2 nitrobenzoate. The samples were incubated in the dark at room temperature for 45 min, and the absorbance was read at 412 nm. GSH concentration was calculated by calibration curves constructed using commercial standards. All reagents were purchased from Sigma-Aldrich (St. Louis, MO, USA). Creatine kinase was measured in a spectrophotometer (Hitachi U-1900, Hitachi, Tokyo, Japan).

### 4.9. Statistical Analysis

Data are presented as mean ± SD. The distribution of all dependent variables was examined by the Shapiro-Wilk test and was found to not differ significantly from normal. Two-way ANOVA (age (young and elderly) × time (before exercise and 48 h post exercise)) with repeated measurements on time were used to analyze isometric peak torque, ROM, DOMS, CK, position sense, protein carbonyls, and GSH. If a significant interaction was obtained, pairwise comparisons were performed through simple main effect analysis. Differences on physical characteristics between young and elderly were examined by unpaired Student’s *t*-test. The level of statistical significance was set at α = 0.05. SPSS version 21.0 was used for all analyses (SPSS Inc., Chicago, IL, USA).

## References

[1] Nair K.S. (2005). Aging muscle. Am. J. Clin. Nutr..

[2] Janssen I. (2006). Influence of sarcopenia on the development of physical disability: The cardiovascular health study. J. Am. Geriatr. Soc..

[3] Metter E.J., Talbot L.A., Schrager M., Conwit R. (2002). Skeletal muscle strength as a predictor of all-cause mortality in healthy men. J. Gerontol. A Biol. Sci. Med. Sci..

[4] LaStayo P., McDonagh P., Lipovic D., Napoles P., Bartholomew A., Esser K., Lindstedt S. (2007). Elderly patients and high force resistance exercise—A descriptive report: Can an anabolic, muscle growth response occur without muscle damage or inflammation?. J. Geriatr. Phys. Ther..

[5] Bauman A., Bull F., Chey T., Craig C.L., Ainsworth B.E., Sallis J.F., Bowles H.R., Hagstromer M., Sjostrom M., Pratt M. (2009). The international prevalence study on physical activity: Results from 20 countries. Int. J. Behav. Nutr. Phys. Act..

[6] Joubert J., Norman R., Lambert E.V., Groenewald P., Schneider M., Bull F., Bradshaw D., South African Comparative Risk Assessment Collaborating Group (2007). Estimating the burden of disease attributable to physical inactivity in south africa in 2000. S. Afr. Med. J..

[7] Paschalis V., Nikolaidis M.G., Theodorou A.A., Panayiotou G., Fatouros I.G., Koutedakis Y., Jamurtas A.Z. (2011). A weekly bout of eccentric exercise is sufficient to induce health-promoting effects. Med. Sci. Sports Exerc..

[8] Hoppeler H. (1986). Exercise-induced ultrastructural changes in skeletal muscle. Int. J. Sports Med..

[9] Lavender A.P., Nosaka K. (2006). Comparison between old and young men for changes in makers of muscle damage following voluntary eccentric exercise of the elbow flexors. Appl. Physiol. Nutr. Metab..

[10] Nosaka K., Newton M., Sacco P. (2002). Delayed-onset muscle soreness does not reflect the magnitude of eccentric exercise-induced muscle damage. Scand. J. Med. Sci. Sports.

[11] Paschalis V., Koutedakis Y., Jamurtas A.Z., Mougios V., Baltzopoulos V. (2005). Equal volumes of high and low intensity of eccentric exercise in relation to muscle damage and performance. J. Strength Cond. Res..

[12] Armstrong R.B. (1990). Initial events in exercise-induced muscular injury. Med. Sci. Sports Exerc..

[13] Paschalis V., Nikolaidis M.G., Giakas G., Jamurtas A.Z., Owolabi E.O., Koutedakis Y. (2008). Position sense and reaction angle after eccentric exercise: The repeated bout effect. Eur. J. Appl. Physiol..

[14] Weerakkody N.S., Percival P., Hickey M.W., Morgan D.L., Gregory J.E., Canny B.J., Proske U. (2003). Effects of local pressure and vibration on muscle pain from eccentric exercise and hypertonic saline. Pain.

[15] Paschalis V., Nikolaidis M.G., Giakas G., Jamurtas A.Z., Pappas A., Koutedakis Y. (2007). The effect of eccentric exercise on position sense and joint reaction angle of the lower limbs. Muscle Nerve.

[16] Paschalis V., Nikolaidis M.G., Theodorou A.A., Giakas G., Jamurtas A.Z., Koutedakis Y. (2010). Eccentric exercise affects the upper limbs more than the lower limbs in position sense and reaction angle. J. Sports Sci..

[17] Wood S.A., Morgan D.L., Proske U. (1993). Effects of repeated eccentric contractions on structure and mechanical properties of toad sartorius muscle. Am. J. Physiol..

[18] Nikolaidis M.G., Jamurtas A.Z., Paschalis V., Fatouros I.G., Koutedakis Y., Kouretas D. (2008). The effect of muscle-damaging exercise on blood and skeletal muscle oxidative stress: Magnitude and time-course considerations. Sports Med..

[19] Nikolaidis M.G., Kyparos A., Dipla K., Zafeiridis A., Sambanis M., Grivas G.V., Paschalis V., Theodorou A.A., Papadopoulos S., Spanou C. (2012). Exercise as a model to study redox homeostasis in blood: The effect of protocol and sampling point. Biomarkers.

[20] Nikolaidis M.G., Paschalis V., Giakas G., Fatouros I.G., Koutedakis Y., Kouretas D., Jamurtas A.Z. (2007). Decreased blood oxidative stress after repeated muscle-damaging exercise. Med. Sci. Sports Exerc..

[21] Theodorou A.A., Nikolaidis M.G., Paschalis V., Koutsias S., Panayiotou G., Fatouros I.G., Koutedakis Y., Jamurtas A.Z. (2011). No effect of antioxidant supplementation on muscle performance and blood redox status adaptations to eccentric training. Am. J. Clin. Nutr..

[22] Theodorou A.A., Nikolaidis M.G., Paschalis V., Sakellariou G.K., Fatouros I.G., Koutedakis Y., Jamurtas A.Z. (2010). Comparison between glucose-6-phosphate dehydrogenase-deficient and normal individuals after eccentric exercise. Med. Sci. Sports Exerc..

[23] Roth S.M., Martel G.F., Ivey F.M., Lemmer J.T., Tracy B.L., Hurlbut D.E., Metter E.J., Hurley B.F., Rogers M.A. (1999). Ultrastructural muscle damage in young vs. Older men after high-volume, heavy-resistance strength training. J. Appl. Physiol..

[24] Lavender A.P., Nosaka K. (2008). Changes in markers of muscle damage of middle-aged and young men following eccentric exercise of the elbow flexors. J. Sci. Med. Sport.

[25] Dedrick M.E., Clarkson P.M. (1990). The effects of eccentric exercise on motor performance in young and older women. Eur. J. Appl. Physiol. Occup. Physiol..

[26] Manfredi T.G., Fielding R.A., O'Reilly K.P., Meredith C.N., Lee H.Y., Evans W.J. (1991). Plasma creatine kinase activity and exercise-induced muscle damage in older men. Med. Sci. Sports Exerc..

[27] Ploutz-Snyder L.L., Giamis E.L., Formikell M., Rosenbaum A.E. (2001). Resistance training reduces susceptibility to eccentric exercise-induced muscle dysfunction in older women. J. Gerontol. A Biol. Sci. Med. Sci..

[28] Proske U., Allen T.J. (2005). Damage to skeletal muscle from eccentric exercise. Exerc. Sport Sci. Rev..

[29] Brockett C., Warren N., Gregory J.E., Morgan D.L., Proske U. (1997). A comparison of the effects of concentric versus eccentric exercise on force and position sense at the human elbow joint. Brain Res..

[30] Bailey D.M., McEneny J., Mathieu-Costello O., Henry R.R., James P.E., McCord J.M., Pietri S., Young I.S., Richardson R.S. (2010). Sedentary aging increases resting and exercise-induced intramuscular free radical formation. J. Appl. Physiol..

[31] Nyberg M., Mortensen S.P., Cabo H., Gomez-Cabrera M.C., Vina J., Hellsten Y. (2014). Roles of sedentary aging and lifelong physical activity in exchange of glutathione across exercising human skeletal muscle. Free Radic. Biol. Med..

[32] Powers S.K., Jackson M.J. (2008). Exercise-induced oxidative stress: Cellular mechanisms and impact on muscle force production. Physiol. Rev..

[33] Dixon C.B., Robertson R.J., Goss F.L., Timmer J.M., Nagle E.F., Evans R.W. (2006). The effect of acute resistance exercise on serum malondialdehyde in resistance-trained and untrained collegiate men. J. Strength Cond. Res..

[34] Sacheck J.M., Decker E.A., Clarkson P.M. (2000). The effect of diet on vitamin e intake and oxidative stress in response to acute exercise in female athletes. Eur. J. Appl. Physiol..

[35] Halliwell B., Gutteridge J. (2007). Free Radicals in Biology and Medicine.

[36] Hawkins C.L., Morgan P.E., Davies M.J. (2009). Quantification of protein modification by oxidants. Free Radic. Biol. Med..

[37] Rushworth G.F., Megson I.L. (2014). Existing and potential therapeutic uses for n-acetylcysteine: The need for conversion to intracellular glutathione for antioxidant benefits. Pharmacol. Ther..

[38] Lees S.J., Booth F.W. (2004). Sedentary death syndrome. Can. J. Appl. Physiol..

[39] Barbat-Artigas S., Rolland Y., Cesari M., Abellan van Kan G., Vellas B., Aubertin-Leheudre M. (2013). Clinical relevance of different muscle strength indexes and functional impairment in women aged 75 years and older. J. Gerontol. A Biol. Sci. Med. Sci..

[40] Daley M.J., Spinks W.L. (2000). Exercise, mobility and aging. Sports Med..

[41] Reddy Y.N., Murthy S.V., Krishna D.R., Prabhakar M.C. (2004). Role of free radicals and antioxidants in tuberculosis patients. Indian J. Tuberc..

[42] Veskoukis A.S., Kyparos A., Paschalis V., Nikolaidis M.G. (2016). Spectrophotometric assays for measuring redox biomarkers in blood. Biomarkers.

